# The Role of Platelets in Cancer Pathophysiology: Focus on Malignant Glioma

**DOI:** 10.3390/cancers11040569

**Published:** 2019-04-22

**Authors:** Sascha Marx, Yong Xiao, Marcel Baschin, Maximilian Splittstöhser, Robert Altmann, Eileen Moritz, Gabriele Jedlitschky, Sandra Bien-Möller, Henry W.S. Schroeder, Bernhard H. Rauch

**Affiliations:** 1 Department of Neurosurgery, University Medicine Greifswald, 17475 Greifswald, Germany; sascha.marx@uni-greifswald.de (S.M.); yungxiao@hotmail.com (Y.X.); msplittstoehser@yahoo.de (M.S.); robert.altmann@stud.uni-greifswald.de (R.A.); sandra.bien@uni-greifswald.de (S.B.-M.); henry.schroeder@uni-greifswald.de (H.W.S.S.); 2 Department of Pharmacology, Center of Drug Absorption and Transport, University Medicine Greifswald, 17487 Greifswald, Germany; eileen.moritz@uni-greifswald.de (E.M.); gabriele.jedlitschky@uni-greifswald.de (G.J.); 3 Department of Transfusion Medicine, University Medicine Greifswald, 17475 Greifswald, Germany; marcel.baschin@uni-greifswald.de

**Keywords:** glioblastoma multiforme, platelet, immune cell interaction, sphingosine-1-phosphate

## Abstract

The link between thrombocytosis and malignancy has been well known for many years and its associations with worse outcomes have been reported mainly for solid tumors. Besides measuring platelet count, it has become popular to assess platelet function in the context of malignant diseases during the last decade. Malignant gliomas differ tremendously from malignancies outside the central nervous system because they virtually never form distant metastases. This review summarizes the current understanding of the platelet–immune cell communication and its potential role in glioma resistance and progression. Particularly, we focus on platelet-derived proinflammatory modulators, such as sphingosine-1-phosphate (S1P). The multifaceted interaction with immune cells puts the platelet into an interesting perspective regarding the recent advances in immunotherapeutic approaches in malignant glioma.

## 1. Introduction

The glioblastoma multiforme (GBM) is the most common malignant brain tumor in adults. The mean overall survival is hardly beyond one year, despite standard treatment consisting of gross total resection of the contrast enhanced tumor segments followed by radiochemotherapy with temozolomide [1,2]. New treatment modalities such as tumor-treating fields (TTF) [3,4] may ultimately result in a survival benefit, and innovative immunotherapeutic approaches might bear the opportunity of curative interventions in the future [5,6,7,8,9,10]. One major problem for a successful systemic immunotherapy in GBM is the highly immunosuppressive tumor microenvironment which prevents the immune system from effectively attacking the tumor [11,12,13]. The predominant immune cell constituent in GBM tissue are tumor-associated macrophages (TAMs) [14,15], which can be derived from both microglia, the resident type of macrophages in the brain, as well as from bone marrow (BM)-derived peripheral monocytes/macrophages [16,17]. Comprehensive investigations from Pyonteck et al. have shown that about 40% of TAMs are of peripheral origin and invade the brain in a platelet-derived growth factor (PDGF)- and hypoxia-inducible factor-1α (HIF-1α)/stromal-cell-derived factor-1 (SDF-1)-dependent manner [18]. Strikingly, platelets are not only a major source of PDGF and of SDF-1α (CXCL12) [19,20], but also play a central role in both tumor angiogenesis [21,22] and in concerting the tumor microenvironment in solid tumors [23]. Platelets release a plethora of growth factors, inflammatory mediators, and chemokines into the microenvironment, and overwhelming evidence reveals platelets as key components in cancer biology and the challenges in targeting platelets for cancer treatment are currently discussed [21]. In comparison, the putative role of platelets in GBM pathophysiology and specifically in affecting immune cell functions such as conversion of macrophages into immunosuppressive TAMs in GBM is less clear to date. 

**Figure 1:**
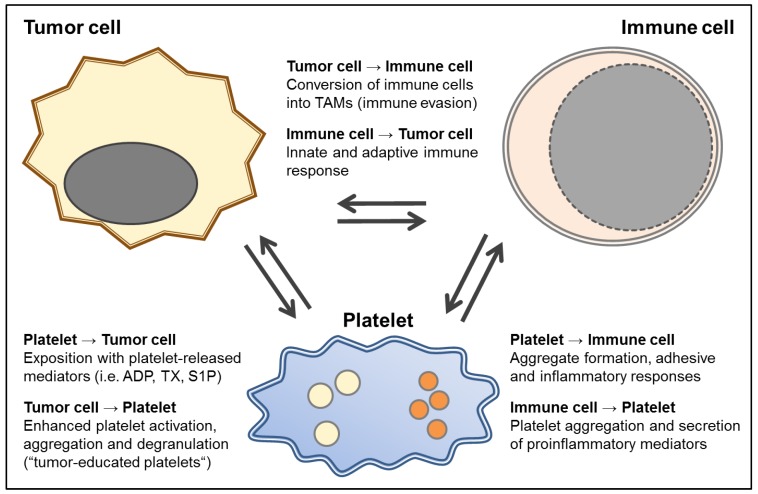
A scheme of the multiple and mutual interactions between tumor and immune cells and platelets. Platelets and tumor cells interact at various levels. Platelet-derived paracrine mediators such as adenosine diphosphate (ADP) as well as lipid signaling mediators like thromboxane (TX) and sphingosine-1-phosphate (S1P) are secreted upon platelet activation and can modulate tumor cell activity [39]. Tumor cells can in turn enhance platelet reactivity and educate platelets to release tumorigenic and angiogenic mediators and stimulate thrombopoiesis [45,46]. While the platelet itself can be seen as an immune cell, it can also interact in multiple ways with the different nucleated immune cells [47,48]. For example, platelet derived mediators such as S1P enhance monocyte activity levels via upregulation of the protease-activated thrombin receptors (PARs)-1 and -4 resulting in enhanced chemotactic capacity and amplifying cyclooxygenase-2 (COX-2) expression [49,50]. In turn, immune cells, such as monocytes, can also release tissue factor-rich microparticles to enhance fibrin formation, enhance clot stability, and ultimately thrombosis [51]. Activated immune cells such as microglia and also peripheral monocytes enter the tumor tissue [15]. In the case of the GBM, this results in the conversion of the infiltrating immune cells into immunosuppressive tumor-associated macrophage (TAMs), which are modulated to protect the tumor. Therapeutic approaches to target these immunosuppressive defense mechanisms are currently under discussion [14,15].

The GBM is not only characterized by its highly infiltrative growth pattern and immunosuppressive properties but it is also a highly prothrombotic tumor entity. Typically, the balance between the procoagulant and anticoagulant system as well as the fibrinolytic system is severely impaired in favor of hypercoagulability in GBM patients [24]. High-grade gliomas, for instance, histologically exhibit areas of necrosis due to anoxia and microthrombosis despite diffuse microvascular proliferation and represent a significant risk factor for thromboembolism. As a consequence, this tumor is associated with a high risk for venous thromboembolism (VTE) and also cardiovascular events [25,26,27]. The heterogeneous morphology of GBM is characterized by its infiltrative growth, intense neoangiogenesis, and pseudopalisading necrosis [28]. In addition, striking endothelial cell proliferation and occlusive intravascular thrombosis can be observed in GBM tissue [29]. VTE is a common complication of cancer and it has been estimated that patients with cancer have a 4-fold to 20-fold increased risk of VTE, which is further accentuated by chemotherapy. The cumulative incidence of symptomatic VTE among patients with glioma has been estimated to be as high as 32.2% during their course of therapy [24,29,30]. Furthermore, inside the GBM a multitude of thrombosed vessels can be seen reflecting the state of hypercoagulability in these patients. However, the survival did not differ between GBM patients with and without VTE and was 53% after 12 months in both groups [31]. Interestingly, recent studies support the assumption that the plasma hypercoagulable profile seen in GBM patients is related to adverse outcomes [24]. Therefore, the significance and prognostic value of characterizing the coagulation profile in GBM patients as a novel approach to an individualized therapy of GBM is under discussion [24]. In addition, platelets release mediators which can directly or indirectly, i.e. by modulating immune responses, modify tumor cell activity, tumor growth and tumor angiogenesis (see Figure 1). The precise contribution of platelet-derived mediators in GBM in this context is not well studied to date.

## 2. Platelets and Solid Tumors

Platelets are well known for their classical function as a key player of the primary hemostasis, but platelets have a much broader range of other functions as well [32]. The relevance of platelets in oncological processes was first described in the 19th century by Leopold Riess and Theodor Billroth [33,34,35]. Malignant tumors have an impact on the platelet number and the functional state of platelets [32]. Platelets can be activated by tumor cells [36]. The daily use of aspirin can diminish the risk of dying due to a visceral malignancy [37,38,39]. Buergy et al. excellently reviewed the role of platelets in malignant diseases, which include platelet enhanced formation of metastasis, platelet-induced angiogenesis, and malignancy-induced thrombocytosis [33]. In recent years, a correlation of thrombocytosis and decreased overall survival could be shown for a magnitude of solid cancers such as lung, colon, breast, esophageal, gastric, renal, ovarian, and melanoma. However, since different tumor types induce thrombopoiesis, this may be an epiphenomenon rather than a causal relation. At least, the possible causality between thrombocytosis and malignancy is not fully understood to date [33]. Interestingly, decreased platelet reactivity in patients with cancer has been associated with a high risk of venous thromboembolism and poor prognosis, presumably as a consequence of continuous activation [40]. Thus, the status of platelets reactivity, which is modulated by solid tumors, appears to be of key relevance [40]. Platelets induce tumor angiogenesis by secreting proangiogenetic cytokines such as vascular endothelial growth factor (VEGF), PDGF, transforming growth factor (TGF), endothelial cell growth factor (ECGF), insulin-like growth factor (ILGF), basic fibroblast growth factor (bFGF, FGF-2), angiopoietin-1 as well as the lipid mediator sphingosine-1-phosphate [33]. Platelets cover circulating tumor cells in a P-selectin-dependent mechanism and thereby support their extravasation and prevent natural killer (NK) cell-mediated tumor cell lysis. Thus, platelets are a key player in the formation of distant metastasis in malignant diseases [41,42]. The inhibition of platelet binding to tumor cells by heparin can reduce the formation of metastasis [43]. Although it is well known that circulating GBM cells are present in patients suffering from GBM and tumor RNA can even be found in circulating platelets of these patients [44], GBM patients virtually never form metastases outside the central nervous system (CNS). This is a fundamental difference to malignancies outside the CNS, where distant metastases are a main determinant for disease progression and patient prognosis. As the CNS provides a unique microenvironment, i.e. determined by the blood–brain barrier and a distinct microglia, the contribution of platelets to GBM pathogenesis may vary from non-CNS tumors and is not well studied in this context. Thus, the functional interactions between platelets and the GBM may differ substantially from the role of platelets in tumors outside the CNS. Figure 1 summarizes the different mutual levels of interaction between tumor cells and platelets, and the role of platelets in concerting immune cell functions. The precise molecular levels of interaction of platelets with glioblastoma cells have to be clarified in future studies.

## 3. Platelets and Glioblastoma

More than 10 years ago, Brockmann et al. showed an association between thrombocytosis and a decreased overall survival in a group of 158 GBM patients [52]. They observed a significantly shorter median survival time in patients with preoperative thrombocytosis of 4 months (95% confidence interval (95% CI), 3–6 months) compared to 11 months survival time (95% CI, 8–13 months; *p* = 0.0006) in patients with normal platelet count [52]. The results suggested that a condition of preoperative thrombocytosis may represent a prognostic factor associated with shorter survival time in patients with glioblastoma. However, in a consecutive investigation involving 140 GBM patients, Lopes at al. could not confirm this observation [53]. Here, the authors found no correlation of neutrophil–lymphocyte ratio, platelets–lymphocyte ratio, or the absolute counts of neutrophils, lymphocytes, and platelets with overall survival in multivariate analyses [53]. Strikingly, the mean overall survival time in this study was 19.4 ± 14.3 months and the mean progression-free survival was 9.4 ± 8.7 months. Different adjuvant treatment regimens, which have changed over time, may have contributed to this difference in survival time. In agreement with this estimation, the rate of combined radiochemotherapy in the earlier study from Brockmann et al. [52] was below 40% in total, while in the more recent study from Lopes at al. [53] a large majority of patients of about 84% were treated according to the combined radiochemotherapy protocol established by Stupp et al. [1]. Besides this, different GBM subtypes exist, which were not further characterized in both of these studies, but should be distinguished. These diverse GBM variants have been described to exhibit substantial heterogeneity, including molecular, histopathological, and genomic features [54,55]. The understanding of the precise molecular pattern of these diverse tumor variants should be improved in the future. Both studies addressing the possible role of platelet count in GBM prognosis did not define the clinical features of molecular variants. Furthermore, the number of included patients—owing to the rare nature of the disease—was rather limited, making these controversial observations rather difficult to interpret. 

Another aspect, which may be of relevance for the pathophysiological impact of platelet functions in GBM development is the extent of platelet reactivity. In a recent study from our group, we could show, for the first time in a homogenous cohort of primary diagnosed GBM patients, that the activation status of circulating platelets is increased [56]. However, in comparison to visceral malignancies, such as colon cancer, the available data on the clinical effects of platelet function inhibitors in GBM, e.g., with aspirin, appear sparse and inhomogeneous. A large epidemiological study with over 300,000 participants did not find any correlation between the intake of aspirin or other NSAID and the development of glioma and/or GBM [57]. However, the assessment of aspirin use was only according to self-questionnaire and there was no monitoring of treatment efficacy, i.e., by measuring thromboxane levels [57]. In addition, the definition of regular aspirin use as intake of more than two doses per week in this study [57] makes it highly unlikely, that aspirin treatment was efficacious in this study. Furthermore, the duration of aspirin treatment in the study was only one year. In comparison, the well documented effects of aspirin in colorectal cancer occur after a treatment time of at least 5 to 10 years [38,39]. To date, the precise underlying mode of action of the long-term effect of aspirin is neither in colorectal cancer nor in other tumor entities fully understood. Especially in colorectal cancer, the antitumor effects of aspirin occur at antiplatelet doses implicating a role of platelets in tumor pathogenesis. However, since many cancer cell types express both isoforms of cyclooxygenase, COX-1 and COX-2, the effects of aspirin may in part also be directly related to the inhibition of COX in cancer cells [37,38,39]. In comparison to the study from Daugherty et al. [57], where aspirin efficacy may be questionable (see above), another more recent study ascertained drug use in glioma patients more thoroughly through the evaluation of drug registries [58]. In their study, Gaist et al. observed that long-term continuous use of low-dose aspirin was associated with an OR (odds ratio) of 0.88 (95% CI: 0.54–1.42) and non-aspirin-NSAID (non-steroidal anti-inflammatory drugs) with an OR of 1.56 (95% CI: 0.71–3.46). According to the authors, their findings may be consistent with a small reduction in glioma risk associated with the long-term use of low-dose aspirin. However, they also point out that their findings should be interpreted cautiously due to limited statistical precision, notably in the duration and dose–response analyses [58]. Taken together, the clinical evidence of the role of platelets both concerning platelet numbers and the use of antiplatelet agents in GBM are limited. Therefore, future and ideally perspective studies are needed to clarify the possible direct role of platelets in GBM. 

## 4. The Immunomodulatory Potential of Platelets in GBM

Besides their role in hemostasis and the above described mechanisms in tumor biology, platelets are well known to interact with the immune system [47,48]. Platelets express Toll-like receptors and, thereby, are sentinel cells for infection [59]. Furthermore, platelets interact in an activity-boosting manner with the complement system [60]. Platelet activation is accompanied with an increased formation of heterotypic platelet–leukocyte conjugates, which is well known as the surrogate of a proinflammatory function and described in inflammatory diseases as sepsis or atherosclerosis [61,62]. In agreement with these (patho)physiological functions, platelet inhibition is known to be anti-inflammatory and, for example, reduces the risk of pulmonary as well as other infections [59,63]. 

Since recently, immunotherapeutical approaches became of interest in GBM patients, the immunomodulatory functions of platelets might be of great interest as well. However, little is known about the role of platelets to modulate immune responses in GBM, yet. For the first time, we could recently show that the formation of heterotypic platelet–monocyte conjugates in the circulation of GBM patients is not increased, although the platelets had an increased activation status in these patients (including an increased P-selectin expression) [56]. This, at first sight a paradoxical finding, may be explained by a reduced expression of PSGL-1 on circulating monocytes in GBM patients [56], since the interaction between platelet P-selectin and monocyte PSGL-1 is the initial and key step in this conjugate formation [61]. In further experiments, we could show that the PSGL-1 expression is tremendously reduced on intratumoral macrophages in GBM as well (unpublished data). Thus, the PSGL-1 phenotype might be a yet unnoted biomarker for the GBM-induced immunosuppression. 

Platelets contain and secrete a multitude of mediators that are known to participate in both hemostasis and inflammation [64]. These mediators predominantly originate from platelet α-granules [65]. Every platelet contains about 50–80 alpha granules. Inside these granules mediators and chemokines, such as P-selectin, CXCL1, platelet factor 4 (PF4) CXCL5, CXCL7, IL-8, and CXCL12, macrophage inflammatory protein-1 (CCL3) and RANTES (CCL5) are stored and can be released upon activation [65]. These mediators play a major role in the regulation of leukocyte migration into tissues and in other proinflammatory functions like phagocytosis, monocyte differentiation, and the generation of reactive oxygen species [66]. Activated platelets release IL-1, which plays a major role in the inflammatory cytokine cascade [67], and TGFβ, which is well known as key mediator of the GBM-induced immunosuppression [68,69,70]. Furthermore, soluble CD40 released from platelets was shown to inhibit regulatory T-cells in a glioma model [71]. As a note, in our own previous studies, we could show a tendency of an increased CD40 expression on platelets in GBM patients [56].

The role of platelets in the pathophysiology of GBM appears, however, to be two-edged. On the one hand, platelet activation may be beneficial to support immune responses, as activated platelets and their secretome can modulate immune responses. Recently, it was shown that an activated platelet-rich plasma clot can inhibit regulatory T-cell migration and prolong overall survival in a GBM model in mice [71]. On the other hand, platelet activation needs to be avoided, since GBM patients have an increased risk for systemic cardiovascular events and the intratumoral occlusion of numerous vessels leads to a hypoxia-induced tumor progression. A further key molecule, which is released from platelets and has been described as a modulator of inflammatory and oncological processes, is sphingosine-1-phosphate (S1P).

## 5. Sphingosine-1-Phosphate in Glioblastoma 

**Figure 2:**
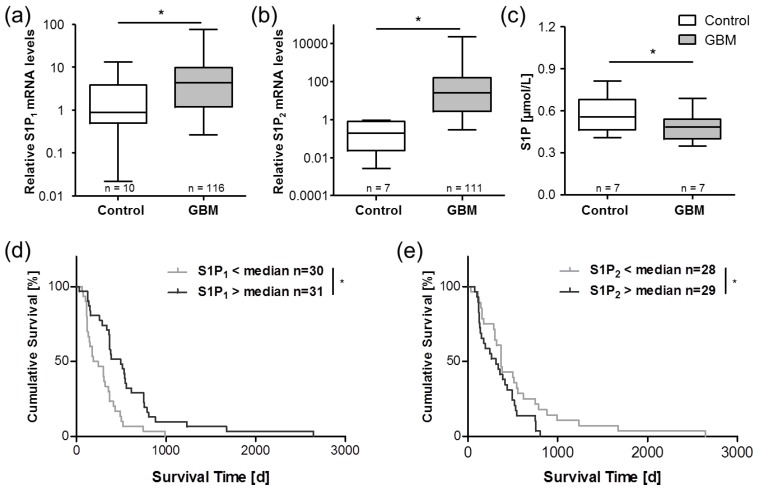
Expression of S1P receptors and circulating peripheral S1P levels are altered in GBM patients. In previous studies, we observed highly upregulated levels of mRNA for the S1P receptors S1P_1_ (**a**) andS1P_2_ (**b**) in GBM tissue [75]. The expression levels of both S1P receptors significantly correlated with the survival time of the respective GBM patients (**d**,**e**). In a later study, our data indicated reduced levels of S1P in platelet-rich plasma of GBM patients (**c**) [56]. These observations point towards a key role of S1P signaling system in the pathophysiology of GBM and are in agreement with studies from other groups [87,88]. Data are adapted from Bien-Möller et al. [75] and Marx et al. [56] and are shown as box plots representing the median as horizontal bars as well as the 5th and 95th percentile. * *p* < 0.05, Mann Whitney U test.

The versatile lipid signaling mediator S1P has emerged as a regulator of a variety of cellular processes including proliferation, metastasis, inflammation, stem cell behavior, and the formation of microvascular networks, which provide nourishment to cancerous cells [72,73,74]. S1P has been recognized in numerous studies over the recent years as an important oncogenic factor involved in multiple cancer entities including breast, colorectal, kidney, lung, melanoma, and GBM [72,75]. Our group could show that the relative amount of S1P secreted from stimulated peripheral platelets of GBM patients is significantly elevated compared to healthy controls [56]. However, the absolute concentration of S1P in platelet-rich plasma of these patients was significantly lower than in the controls (Figure 2C) [56]. Furthermore, a significantly elevated S1P concentration was found in GBM tissue in comparison to the control brain specimens [76], and glioma cells, as well as GBM stem cells, are able to produce and release S1P [77,78]. Since S1P directs immune cell migration via concentration gradient-dependent mechanisms, a reduced peripheral, and in turn, elevated central S1P level in GBM tissue might foster monocyte migration from the peripheral blood into the brain. In addition, S1P has been reported as a key player in the transformation of intratumoral macrophages into ultimately immunosuppressive TAMs via S1P receptor-1 [79]. Thus, reduced peripheral blood S1P levels may drive monocyte invasion into the tumor tissue, and elevated S1P concentrations within the GBM consecutively could support intratumoral conversion of macrophage/microglia into TAMs (see also Figure 1). In addition, S1P is known as mediator between blood coagulation, platelets, and vascular inflammatory responses [49]. Not only the secretion of platelet-derived S1P can be stimulated by coagulation factors such as thrombin [80], but also enzymes which generate S1P in the vasculature can be upregulated by coagulation factors such as the activated factor-X (FXa) [81].

In general, the synthesis of sphingolipid and S1P in particular is tightly controlled by the metabolism of ceramide. Biosynthesis of cellular ceramide derives either de novo from serine, palmitoyl-CoA, and fatty acid, or from the breakdown of membrane-resident sphingomyelin [72,82,83]. S1P is ultimately generated by phosphorylation of sphingosine by the two isoenzymes sphingosine kinase-1 and -2 (SphK1 and SphK2) [49,82,83]. S1P degradation is achieved via dephosphorylation by two S1P-specific phosphatases (SGPP1 and SGPP2) or an irreversible hydrolysis by S1P lyase (SGPL) [84,85]. The formation of concentration gradients is essential in regulating the physiological effects of S1P in vivo [84]. The balance between S1P generation and degradation is also critical for the regulation of cell growth and plays a key role in pathological processes such as carcinogenesis [72,85]. Thus, the inhibition of S1P synthesis results in a sensitization of glioma stem cells against temozolomide as the standard chemotherapeutic in GBM treatment [78]. Interestingly, fingolimod (FTY720)—a sphingosine analogue approved for the treatment of multiple sclerosis—reduced the intracranial growth of brain tumors in a mouse model of GBM [86]. 

Besides elevated basal platelet activation and altered circulating peripheral S1P levels (see Figure 1), a severe dysregulation of the S1P signaling system was also found in tumor tissue samples of GBM patients in previous investigations [75]. Specifically, the S1P receptors S1P_1_ and S1P_2_ (Figure 2) and the S1P-generating enzyme, SPHK1, were significantly upregulated in GBM samples. Intriguingly, the expression levels of both S1P_1_ and S1P_2_ correlated significantly with patient survival time but in divergent ways. In GBM patients with high S1P_1_ mRNA levels, a prolonged survival was observed, while patients with a high S1P_2_ mRNA expression exhibited a shorter survival time [75]. In agreement with the literature, our results from this study implicated a complex interplay between S1P receptors, S1P signaling, and other tumor-promoting signaling cascades in GBM, i.e., opposing functions of S1P_1_ and S1P_2_ in the regulation of cell migration and proliferation. Going forward, a better understanding of the pathological basis of GBM tumors could lead to better diagnostic and treatment protocols, so that a tailored monitoring of platelet-derived proinflammatory modulators such as S1P could be easily incorporated into multiplex biomarker panels and guide clinicians in developing novel immunotherapeutic approaches to gliomas. Prospectively, a pharmacological modulation of S1P levels and/or its receptors may also represent a potential future therapeutic concept in GBM therapy.

## 6. Summary and Conclusions

In summary, the contribution of platelets in tumor development, invasiveness, malignancy, and metastasis is widely acknowledged in the literature. However, the specific contribution of platelets to tumor pathophysiology in GBM is less clear. The preoperative platelet count has been suggested as a potential outcome predictor. However, the currently available data are inconclusive and future studies are needed to gain further insights into the role of platelet number as a possible biomarker. Studies on the reactivity level of platelets in GBM are also sparse to date. Our own data indicate elevated levels of P-selectin and impaired relative responsiveness to platelet stimulation ex vitro, possibly pointing towards an altered immunoregulatory function of platelets during GBM growth and progression. Since thrombosis is a hallmark of GBM, suitable antithrombotic and antiplatelet concepts may be a valuable addition to future individualized targeted therapeutic concepts.
